# An overview of anti-Hepatitis B virus flavonoids and their mechanisms of action

**DOI:** 10.3389/fcimb.2024.1356003

**Published:** 2024-02-29

**Authors:** Malihe Naderi, Zahra Salavatiha, Urvashee Gogoi, Alireza Mohebbi

**Affiliations:** ^1^ Department of Microbiology & Microbial Biotechnology, Faculty of Life Sciences and Biotechnology, Shahid Beheshti University, Tehran, Iran; ^2^ Department of Virology, School of Medicine, Iran University of Medical Sciences, Tehran, Iran; ^3^ Department of Pharmaceutical Sciences, Dibrugarh University, Dibrugarh, Assam, India; ^4^ Vista Aria Rena Gene Inc., Gorgan, Golestan, Iran

**Keywords:** flavonoids, Hepatitis B virus, antiviral therapy, herbal medicine, natural compounds

## Abstract

Flavonoids, a diverse group of polyphenolic compounds found in various plant-based foods, have garnered attention for their potential in combating Hepatitis B Virus (HBV) infection. Flavonoids have demonstrated promising anti-HBV activities by interfering with multiple stages of the HBV life cycle, making them promising candidates for novel antiviral agents. Certain plant families, such as *Theaceae*, *Asteraceae*, *Lamiaceae*, and *Gentianaceae*, are of particular interest for their flavonoid-rich members with anti-HBV activities. Evidences, both *in vitro* and *in vivo*, supports the anti-HBV potential of flavonoids. These subsets of compound exert their anti-HBV effects through various mechanisms, including inhibiting viral entry, disrupting viral replication, modulating transcription factors, enhancing the immune response, and inducing autophagy. The antioxidant properties of flavonoids play a crucial role in modulating oxidative stress associated with HBV infection. Several flavonoids like epigallocatechin gallate (EGCG), proanthocyanidin (PAC), hexamethoxyflavone, wogonin, and baicalin have shown significant anti-HBV potential, holding promise as therapeutic agents. Synergistic effects between flavonoids and existing antiviral therapies offer a promising approach to enhance antiviral efficacy and reduce drug resistance. Challenges, including limited bioavailability, translation from preclinical studies to clinical practice, and understanding precise targets, need to be addressed. Future research should focus on clinical trials, combination therapies, and the development of flavonoid derivatives with improved bioavailability, and optimizing their effectiveness in managing chronic HBV infections.

## Introduction

Infection with the Hepatitis B Virus (HBV) is a major global health concern with far-reaching consequences. It is a huge public health concern because it causes acute and chronic liver damage and is the root cause of hepatocellular carcinoma (HCC), one of the deadliest cancers in the world (Mohebbi et al., 2016, 2018). HBV is a partly double-stranded DNA virus that is spread through contact with contaminated blood and other bodily fluids, making it a highly contagious infection. More than 2 billion individuals globally are anticipated to have been exposed to HBV, with 300 million people living with chronic HBV (CHB) infections by 2023 (Jeng et al., 2023). Chronic HBV infection has a global impact, contributing to a significant morbidity and mortality burden. It is a leading cause of liver cirrhosis and death from liver disease (Martyn et al., 2023; Wang et al., 2023). Notably, HBV is responsible for roughly 887,000 fatalities per year, primarily owing to cirrhosis and HCC, making it a serious concern for world health. The Western Pacific and African areas experience the largest proportion of HBV-related mortality (Hyun Kim and Ray Kim, 2018; Amponsah-Dacosta, 2021; Hsu et al., 2023). This scenario is further complicated by the prevalence of perinatal and vertical HBV transmission from infected mothers to their children, which maintains the infection throughout generations (di Filippo Villa and Navas, 2023; Naderi et al., 2023a, 2023b, 2023c). This is especially troublesome in areas with high incidence and low immunization coverage (Al-Amleh, 2020).

The limitations of conventional antiviral therapy in dealing with HBV is becoming increasingly clear. While therapeutics are available for managing HBV, such as nucleos(t)ide analogues like lamivudine, adefovir, telbivudine, tenofovir, and entecavir, they may require long-term usage and may give rise to the development of drug-resistant viral strains (Rezanezhadi et al., 2019; Mohebbi et al., 2021). In this scenario, search for new potential antiviral agents has focused on natural chemicals (Mohebbi et al., 2022, 2023). Plant-derived compounds have a long history of medicinal and therapeutic use, and up-to-date scientific studies have begun to uncover their antiviral properties. These compounds offer several benefits, including a wide range of structural diversity, well-established safety profiles, and the capacity to target different phases of the viral lifecycle, limiting the possibility of developing resistance (Behl et al., 2021; Abookleesh et al., 2022).

Flavonoids represent a diverse group of polyphenolic compounds found in various plant-based foods, including fruits, grains, vegetables, and beverages. These compounds have been of particular interest due to their well-documented antioxidant, anti-inflammatory, and immunomodulatory properties (Panche et al., 2016; Dias et al., 2021; Bié et al., 2023). Their diverse chemical structures provide them exceptional versatility and the ability to interact with different targets in the viral lifecycle. Flavonoids have been found to interfere with several phases of the viral lifecycle, including viral entrance (Tsukuda et al., 2017), replication (Xu et al., 2020), and assembly (Ninfali et al., 2020). Their mechanisms of action are often multifaceted, making them intriguing candidates for antiviral therapy. Given the global impact of HBV infection and the need for alternative antiviral strategies, this comprehensive review explores the potential of flavonoids as agents to interfere HBV replication and, potentially, alleviate the associated complications. Furthermore, this review study describes the types and sources of flavonoids, the mechanism(s) by which they exert their antiviral activities, and the experimental and clinical data that supports their prospective use as anti-HBV candidate(s).

## Main text

### Search strategy

The search methodology used in this study included a thorough evaluation of the existing literature on flavonoids that are effective agents against HBV infection. A systematic search approach was executed across several databases, including Google Scholar, PubMed, Scopus, and Web of Science, using appropriate terms, including “flavonoids,” “HBV” or “Hepatitis B virus” and “antiviral.” Articles, reviews, and research published prior to the date of the search were evaluated for inclusion. The inclusion criteria were studies that investigated the effect(s) of flavonoids on HBV at the molecular, cellular, animal model, and clinical trial levels. Exclusion criteria included non-English research, studies unrelated to flavonoids or HBV, and studies with insufficient relevance to the subject of this study. No precise publication date was used, and studies with related information were retrieved. Following the selection of relevant literature, data on biological activities of flavonoids, modes of action, chemical origin, and any related experimental or clinical evidence were extracted. The chemical structures of anti-HBV flavonoids were obtained from original research publications and databases.

### Flavonoids: types and sources

Flavonoids are a diverse group of polyphenolic compounds found in a wide range of plant-based foods. They are characterized by a common structure composed of two aromatic rings (A and B) connected by a three-carbon chain that forms an oxygenated heterocycle (C ring) (Dias et al., 2021). This structural diversity has led to the classification of flavonoids into several distinct subclasses, including flavones, flavonols, flavanones, isoflavonoids, anthocyanins, and Chalcones (Panche et al., 2016; Chen et al., 2023).

Citrus fruits, particularly oranges and grapefruits, are rich sources of flavanones. Naringenin and hesperetin are well-known flavanones, and they have been associated with antioxidant and anti-inflammatory properties (Alam et al., 2014; Khan et al., 2020). Flavan-3-ols, also known as flavanols, feature a double bond between C2 and C3. These compounds are prevalent in various plant-derived foods, particularly in fruits like apples, apricots, and cherries, and in beverages such as tea (Hollman et al., 2000; Chen et al., 2023). Catechin and epicatechin are two common flavan-3-ols known for their antioxidant and cardiovascular health-promoting effects (Bernatova, 2018). Furthermore, Flavonols are characterized by a double bond between C2 and C3 in the C ring, similar to flavan-3-ols, but with an added 3-hydroxyl group. Onions, apples, and grapes are examples of dietary sources rich in flavonols. Quercetin, a well-studied flavonol, is recognized for its antioxidant and anti-inflammatory properties (Zhang et al., 2020). In addition, Anthocyanidins are water-soluble flavonoids responsible for the vibrant red, blue, and purple colors in many fruits and vegetables. Berries, red grapes, and red cabbage are examples of foods rich in anthocyanidins (Khoo et al., 2017). Cyanidin, delphinidin, and malvidin are some prominent anthocyanidins that have demonstrated antioxidant and anti-inflammatory effects (Merecz-Sadowska et al., 2023). Flavones are another flavonoid subclass featuring a double bond between C2 and C3 in the C ring. These compounds are often found in leafy green vegetables like spinach, and in spices such as parsley and celery. Apigenin and luteolin are common flavones known for their potential anti-inflammatory and anticancer properties (Salehi et al., 2019; Do Socorro Chagas et al., 2022). Also, isoflavones, notably genistein and daidzein, are predominantly found in soy-based products such as tofu, soy milk, and tempeh. These compounds have a structure resembling 17β-estradiol, the primary female sex hormone, and are classified as phytoestrogens (Alshehri et al., 2021; Kim, 2021). Understanding the diverse classes of flavonoids and their sources is vital in harnessing the potential health benefits of these natural compounds. The presence of flavonoids in various plant-based foods underscores the importance of a balanced and colorful diet in promoting overall health and well-being. In terms of antiviral properties, these chemicals provide interesting options for explore and therapeutic development, as evidenced by their well-documented anti-HBV activities.

#### Plants and their flavonoid constituents with potential anti-HBV activities

The potential herbal medicine and plant-derived constituents on HBV life cycle have been studied (Mohebbi et al., 2018; Indrasetiawan et al., 2019; Roy et al., 2022). In the ongoing search of effective antiviral therapies against HBV, flavonoids have emerged as promising candidates due to their versatile antiviral properties. The understanding of the significance of plants and their families, particularly those rich in flavonoids is very important. These flavonoids have demonstrated promising anti-HBV activities in various *in vitro* and *in vivo* models. These natural substances provide a potential opportunity for the development of new antiviral treatments. This study reviews the potential of several flavonoids that have been found to interfere with the HBV life cycle at different stages. This enhances the prospect of producing innovative antivirals.

**Figure 1:**
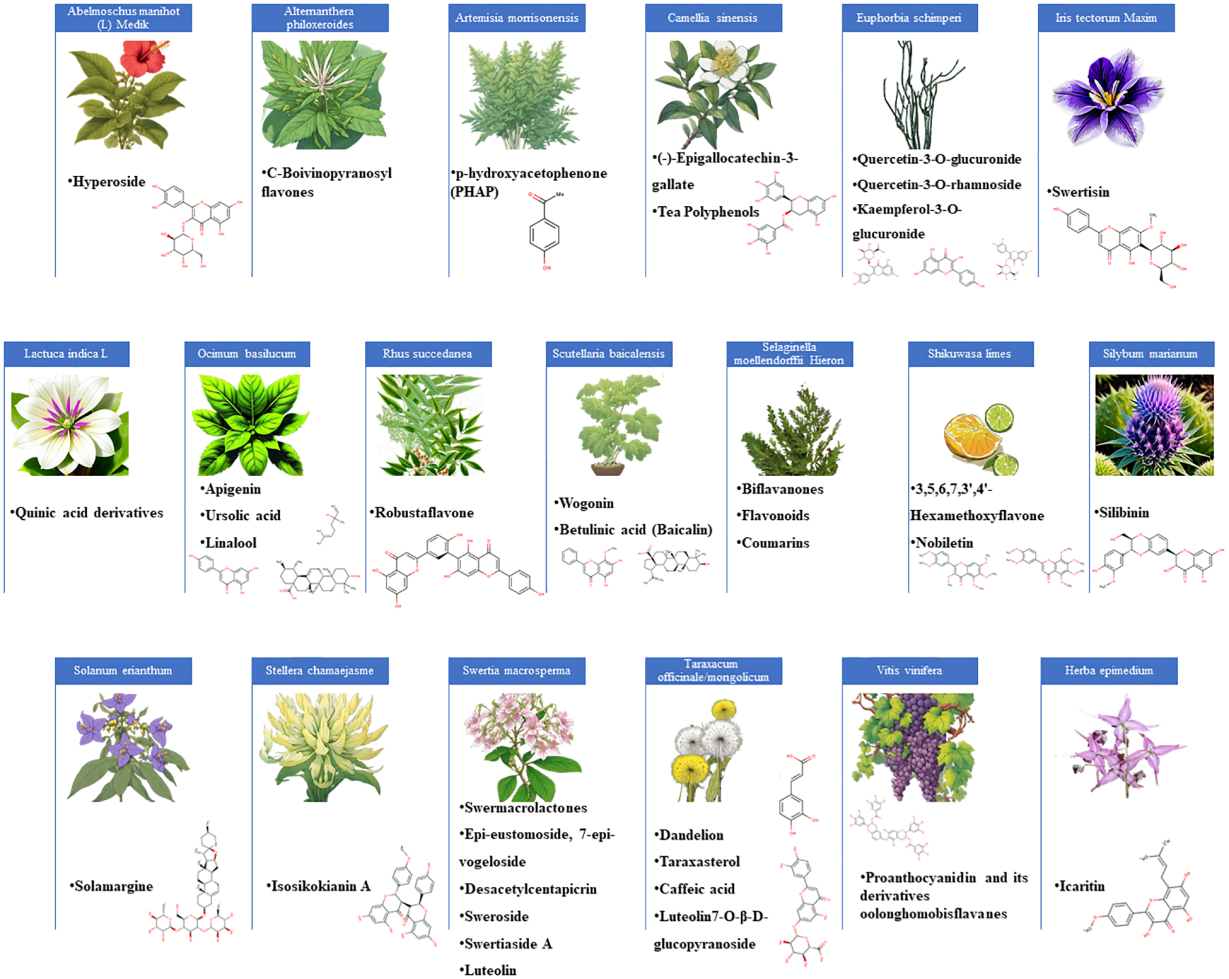
An illustration of various plants known for their flavonoid constituents with potent anti-HBV activities.

**Figure 1** represent the plants and their described flavonoid constituents with active anti-HBV activities. The most frequently reported plant family was *Lamiaceae*, and its species contains promising flavonoids (wogonin and apigenin) and triterpenoids (ursolic acid and betulinic acid). Further reported plant families were included *Theaceae*, home to *Camellia sinensis*, which yields (-)-Epigallocatechin-3-gallate (EGCG) with potent anti-HBV activity (Huang et al., 2014a). Further exploration of this family might reveal additional members with flavonoids sharing similar properties. The *Asteraceae* family, represented by Dandelion (*Taraxacum officinale*), also contains flavonoids with anti-HBV activities (Yang et al., 2020). The *Lamiaceae* family, represented by *Scutellaria baicalensis*, has provided flavonoids like baicalin and wogonin, both having anti-HBV activities (Guo et al., 2007; Huang et al., 2000). The *Gentianaceae* family, hosting *Swertia macrosperma* and its anti-HBV swermacrolactones, holds potential for revealing novel flavonoid compounds with antiviral potential (Wang et al., 2013). The scientific examination of these plant families includes phytochemical analysis, biological tests, computational approaches, and clinical validation, all with the goal of advancing antiviral research and providing hope to individuals suffering by HBV infections. **Table 1** provides details on the plants and their flavonoid compounds.

**Table 1:** Active flavonoids with the anti-HBV activities along with their isolation source.

Compound Name	Plant Source	Plant Family	References
(-)-Epigallocatechin-3-gallate	*C. sinensis*	*Theaceae*	(Huang et al., 2014a)
Proanthocyanidin and its derivatives oolonghomobisflavanes	*Vitis vinifera*	*Vitaceae*	(Tsukuda et al., 2017)
3,5,6,7,3’,4’-Hexamethoxyflavone and Nobiletin	*Shikuwasa* *Limes* (Citrus)	*Rutaceae*	(Hu et al., 2020; Tan et al., 2021)
Swertisin	*Iris tectorum Maxim*	*Iridaceae*	(Xu et al., 2020)
Wogonin	*Scutellaria baicalensis*	*Lamiaceae*	(Huang et al., 2000; Guo et al., 2007)
Isoflavone analogs (Compound 8f)	Not specified	Not mentioned	(Zhang et al., 2013)
Solamargine	*Solanum erianthum D. Don*	*Solanaceae*	(Chou et al., 2012)
Apigenin, Ursolic acid, and linalool	*Ocimum basilucum*	*Lamiaceae*	(Chiang et al., 2005)
p-hydroxyacetophenone (PHAP)	*Artemisia morrisonensis*	*Asteraceae*	(Huang et al., 2014b)
Betulinic acid (Baicalin)	*Scutellaria baicalensis*	*Lamiaceae*	(Huang et al., 2017; Chirumbolo, 2018; Xia et al., 2020)
Isosikokianin A	*Stellera chamaejasme*	*Thymelaeaceae*	(Yang and Chen, 2008)
Biflavanones, Flavonoids, and Coumarins	*Selaginella moellendorffii Hieron*	*Selaginellaceae*	(Cao et al., 2010)
Compounds 4(a-p) (Baicalein derivatives)	Not specified	Not mentioned	(Ma et al., 2017)
Silibinin	*Silybum marianum*	*Asteraceae*	(Lv et al., 2021)
Icaritin	*Herba epimedium*	*Berberidaceae*	(Zhang et al., 2016)
Dandelion (and Taraxasterol)	*Taraxacum officinale*	*Asteraceae*	(Yang et al., 2020)
Hyperoside	*Abelmoschus manihot (L) Medik*	*Malvaceae*	(Wu et al., 2007; Shen et al., 2016)
Quercetin	Various plant sources	Various plant families	(Cheng et al., 2015; Parvez et al., 2021)
Quinic acid derivatives	*Lactuca indica L.*	*Asteraceae*	(Kim et al., 2007)
Silibinin	*Silybum marianum*	*Asteraceae*	(Ghasemi et al., 2013)
Quercetin-3-O-glucuronide (Q3G), Quercetin-3-O-rhamnoside (Q3R), and Kaempferol-3-O-glucuronide (K3G)	*Euphorbia schimperi*	*Euphorbiaceae*	(Parvez et al., 2021)
Robustaflavone hexaacetate	*Rhus succedanea* (*Toxicodendron succedaneum*)	*Anacardiaceae*	(Zembower et al., 1998)
Rosmarinic acid	Not specified	Not mentioned	(Tsukamoto et al., 2018)
Caffeic acid and Luteolin7-O-β-D-glucopyranoside	*Taraxacum mongolicum*	*Asteraceae*	(Jia et al., 2014)
Swermacrolactones A, B, and C, Epi-eustomoside, 7-epi-vogeloside, Desacetylcentapicrin, Sweroside, Swertiaside A, Luteolin, and Isovitexin	*Swertia macrosperma*	*Gentianaceae*	(Wang et al., 2013)
C-Boivinopyranosyl Flavones	*Alternanthera philoxeroides*	*Amaranthaceae*	(Li et al., 2016)

### Mechanisms of antiviral effects of flavonoids

Several flavonoids with significant anti-HBV activity have been reviewed in the present study (**Table 2**). Compounds showed different mechanisms of action that make them potential candidates for further research and drug development. Here, the mechanisms by which flavonoids exert their anti-HBV actions are addressed. Accordingly, the biological activities of flavonoids, *in vitro* and *in vivo* data, clinical insights, synergistic effects with existing antiviral medicines, challenges, and future approaches are all reviewed.

**Table 2:** The mechanism(s) of actions of flavonoids on different aspects of HBV *in vitro* or *in vivo*.

Compound Name	Mechanisms of Anti-HBV Activity	Biological Activities	Synergy
EGCG	- Impairing virus endocytosis/cell fusion. -Impairs clathrin-mediated endocytosis.	10 μM	
EGCG in Human liver chimeric mice	- Decrease in rcDNA and HBsAg mRNA. - Inhibited the expression of fah and HBcAg.	10 μM	–
EGCG via Farnesoid X Receptor Alpha	- EGCG interacts with the LBD of FXRa. - Targeting transcription factors (RXRa).	100 μM GTCs for HBsAg and HBeAg (99% inhibition rate)	–
EGCG in HBV-induced autophagy	- Induces autophagosome formation and inhibits HBV replication.	–	–
PAC analogue OHBF-C	- Blocks viral attachment to NTCP. - Blocks preS1-NTCP interaction. - Reduces HBsAg, HBeAg, HBcAg, and cccDNA levels. - Reduces infectivity of HBV particles.	4.3 ± 1.2 µM	
Hex	- Reduces intracellular HBV RNAs and DNA *in vivo*. - Inhibition of HBV promoters. - HNF3α-mediated transcriptional inhibition.	11.37 μM	ETV
Swertisin	- Reduces HBsAg and HBeAg levels *in vitro*. - Reduces secreted and intracellular HBV DNA. - Inhibits HBV replication.	5 μM	
Wogonin	- Reduces secretion of HBsAg and HBeAg. - Reduces extracellular HBV DNA levels. - Inhibits DHBV DNA polymerase activity in ducks. - Reduces serum HBV DNA levels in human HBV-transgenic mice.	2.56 μM, 4 μM, and 0.57 μM for HBsAg, HBeAg, and HBV DNA, respectively	
Isoflavone analogs	Compound 8f exhibited strong anti-HBV activity.	10.22 mM, 4.07 mM, and 2.34 mM for HBsAg, HBV DNA, and HBeAg, respectively.	
Solamargine	- Inhibits HBsAg and DNA replication.	IC_50_ of 1.57 μM and 2.17 μM for HBsAg and HBD DNA.	
Apigenin	- Apigenin, ursolic acid, and linalool show potent anti- HBsAg and HBeAg effects.	7.1 mM and 12.8 mM for HBsAg and HBeAg, respectively.	3TC & glycyrrhizin
PHAP	- Induces endoplasmic reticulum stress. - Inhibit supernatant HBsAg and HBeAg secretion, and HBV DNA level. - Affect HBV viral particle secretion through ER stress. - Increases HBsAg expression by directly acting on preS promoter. - Interrupts HBV maturation by disrupting GRP78 gene chaperone expression.	294.1 μM	3TC
Baicalin and flavocoxid	- Baicalin inhibits HBV viral RNAs. -Baicalin induce a pro-inflammatory response. - Involve IL6, histone acetylation, and inhibition of viral genome replication.	50 μM	ETV
Compounds Isolated from *S. chamaejasme*	- Compound 1 (Isosikokianin A) is a flavonoid with structural diversity. - Compounds reported to have antiviral effects against HBV.	0.2 μM	3TC
4K	- Targets various stages of HBV life cycle. - 4c inhibited the expression of HBeAg and HBsAg. - Influence host cellular factors (Heme oxygenase-1 (HO-1)).	50 to 100 μM	
Silibinin	- Inhibits cell growth. - Suppresses MMPs in cell lines.- - Inhibits HBV DNA and proteins. - Down-regulation of hepatocyte nuclear factors (HNF1α, and HNF4α).	–	ETV
Icaritin	- Inhibits AFP expression mediated by miRNAs. - Multiple pathways of inhibiting tumor cell proliferation (IL-6/Jak2/Stat3). - Triggers the mitochondrial/caspase apoptotic pathway	10 μM	–
Dandelion and taraxasterol	- Inhibits HBsAg and HBeAg secretion. - Inhibits HBV DNA. - Decrease protein levels of PTBP1 and SIRT1	100 μM for Dandelion and 48 μM for Taraxasterol	–
Hyperoside	- Inhibits HBeAg and HBsAg expression. - Inhibits DHBV-DNA in duckling model. - Improves hepatocellular architecture and reduces necrosis.	~0.012-0.015 g/L and 0.009-0.011 g/L for HBeAg and HBsAg, respectively	–
Quercetin and kaempferol	- Inhibits HBsAg and HBeAg expression. - Reduces intracellular and extracellular viral DNA levels. - Exhibits potential for sustained anti-HBV activity.	22.3−23.5 μM	3TC
Quercetin and Rosmarinic Acid	- Inhibit ϵ-Pol binding and reduces HBV progenies.	30 μM for RA	–
*Taraxacum mongolicum* extract	- Exhibits hepatoprotective effects and inhibits HBsAg, HBeAg, and DNA.	10-100 µg/ml for HBsAg and 50-100 µg/ml for HBeAg and HBV DNA	–

**Figure 2:**
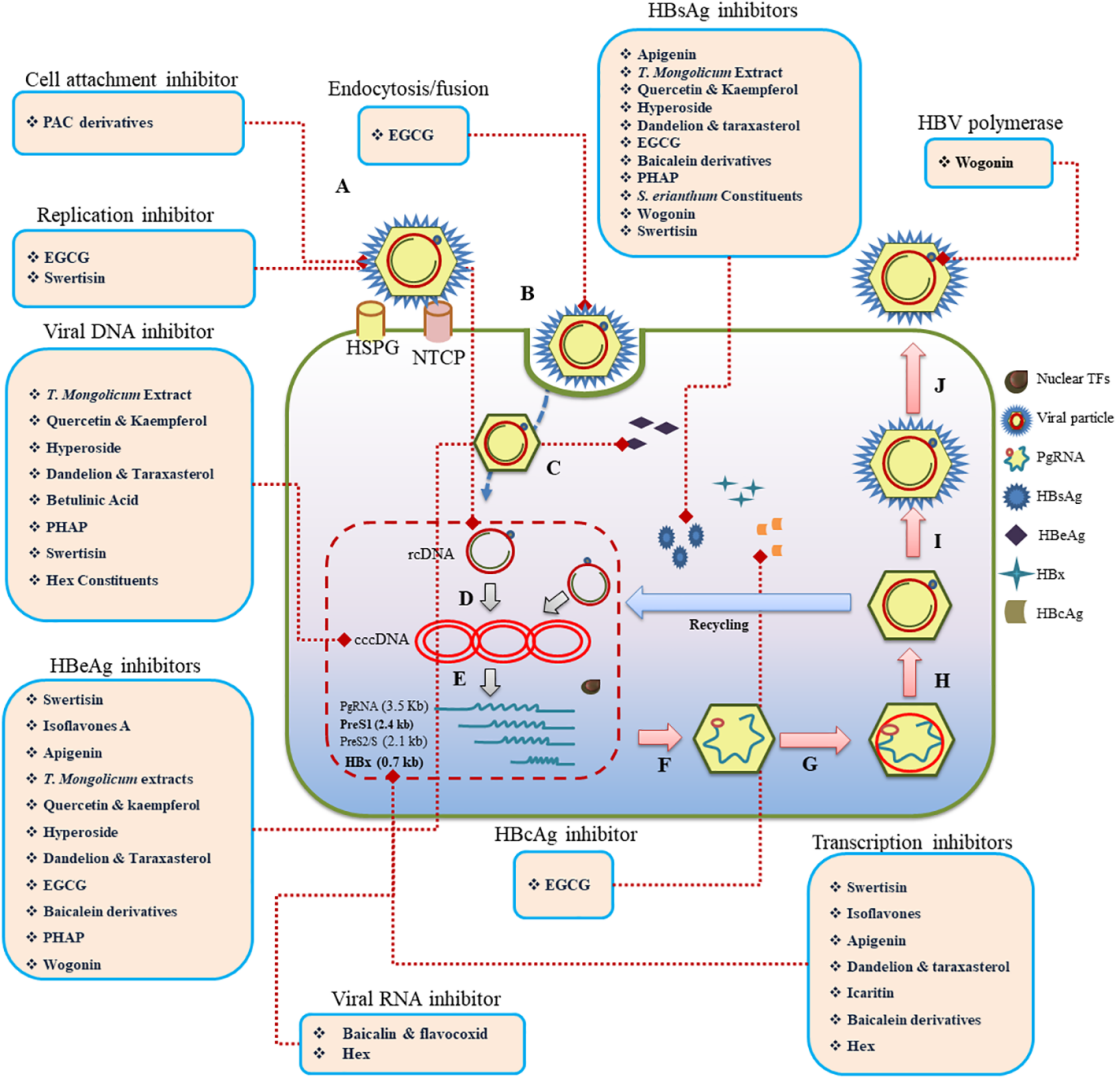
Schematic representation of the life cycle, highlighting key stages from viral attachment to budding **(A–J)**. Flavonoids acting on various stages of the process of HBV infection are provided within rectangles. Nuclear TFs, Nuclear transcription factors; Pg RNA, pregenomic RNA; HBsAg, HBV surface antigen; HBeAg, HBV accessory “e” antigen; HBx, HBV transactivating protein X; HBcAg, HBV core antigen; rcDNA, relaxed circular DNA; cccDNA, covalently closed circular DNA; HSPG, heparan sulphate proteoglycan; NTC, N taurocholate co-transporting polypeptide.

Flavonoids exhibit a variety of antiviral mechanisms (**Figure 2**), which contribute to their potential against HBV. One of the key mechanisms include **i**nhibition of viral entry. Accordingly, EGCG and proanthocyanidin (PAC) suppress HBV infection by interfering with the virus’s endocytosis and cell fusion, respectively (He et al., 2011; Tsukuda et al., 2017). They block viral attachment to specific cell surface receptors, Na^+^ taurocholate co-transporting polypeptide (NTCP), thereby preventing viral entry, resulting in reduction of HBV antigens and DNA. EGCG is one of the most studied flavonoids, and its anti-HBV mechanisms of action are explored thoroughly. In this context, EGCG, a green tea ingredient, suppresses HBV infection at different viral state of replication, including entry (Huang et al., 2014a), DNA synthesis (He et al., 2011), gene expression (Wang et al., 2020), and replication (He et al., 2011). It primarily hinders the entry of HBV into hepatocytes by impairing the virus’s interaction with NTCP, clathrin-mediated endocytosis, and cell fusion steps at 10 μM concentration in a dose- and time-dependent manner (Huang et al., 2014a). EGCG is more effective in inhibiting HBV infection compared to other green tea catechins. It can also inhibit HBV infection when added during the inoculation process. Furthermore, EGCG reduces HBV infection by decreasing HBV cccDNA and mRNA levels, affecting core and HBsAg protein levels (Huang et al., 2014a). It also reduces HBV DNA synthesis, but does not inhibit HBV replication, assembly, or release, suggesting its anti-HBV mechanism primarily targets the synthesis of HBV DNA through interfering viral entry. EGCG has also been found to suppress the expression of HBeAg and HBsAg, downregulate preCore mRNA levels, and inhibit HBV core promoter activity at 10 μM concentration (He et al., 2011) EGCG has been demonstrated to reduce HBsAg and HBeAg (99% inhibition rate) in a dose-dependent manner by targeting the transcription factor Farnesoid X Receptor Alpha (FXRa), indicating its potential as an anti-HBV medication (Xu et al., 2016). EGCG’s therapeutic effect on HBV infection is supported by *in vivo* studies (Lai et al., 2018), indicating its promise as an antiviral agent. Additionally, EGCG can induce autophagosome formation and opposes HBV-induced incomplete autophagy, further contributing to its antiviral effects.

Flavonoids further exerts their antiviral effects on viral replication. Compounds, including baicalin and betulinic acid (BA), inhibit the replication of HBV viral RNAs, disrupting viral replication and reducing the production of new virions. Modulation of transcription factors is another mechanism by which compounds like wogonin (Huang et al., 2000) and rosmarinic acid (Tsukamoto et al., 2018) influence host cellular factors related to HBV replication. They target hepatocytes’ protein, ϵ-Pol (Tsukamoto et al., 2018), affecting viral transcription. Flavonoids are well-known for their antioxidant properties, which play a crucial role in modulating oxidative stress associated with HBV infection. By reducing the levels of reactive oxygen species (ROS) and inhibiting lipid peroxidation, flavonoids help protect hepatocytes and liver tissues from oxidative damage. These antioxidant effects can contribute to the overall reduction in liver inflammation and damage associated with CHB infection. Enhancement of immune response by flavonoids, like baicalin, induce an endogenous pro-inflammatory response to HBV, producing an autocrine IFN-γ reaction and promoting the expression of IFN α/β and IFN-γ (Chirumbolo, 2018), contributing to the host cell’s antiviral response. Further details are provided in **Table 2**.

### Experimental evidences of anti-HBV effects of flavonoids

Several studies have emphasized flavonoids’ anti-HBV potential. These studies mainly employed both *in vitro* and *in vivo* methods to assess the efficiency of flavonoids against HBV. *In vitro* research has been conducted to investigate the mechanisms by which flavonoids suppress HBV. Flavonoids have been to decrease HBsAg and HBeAg secretion shown in studies using hepatoma cell lines (HepG2.2.15 and HuH-7), as well as to decrease viral RNA and intracellular/extracellular HBV DNA levels in a dose- and time-dependent manner (Guo et al., 2007; Huang et al., 2017; Xu et al., 2020; Tan et al., 2021). Furthermore, molecular docking analysis have revealed that flavonoids can interact with viral proteins, transcription factors, and nucleotides, potentially interfering with viral replication (Parvez et al., 2021).

Animal models have offered critical insights into the flavonoids’ anti-HBV properties. *In vivo* tests using human liver chimeric mice (Lai et al., 2018), ducks (Guo et al., 2007), and infected ducklings (Wu et al., 2007) have shown that flavonoids such as EGCG, hyperoside, and quercetin significantly inhibit HBV replication, resulting in decreased viral DNA and antigen levels. These studies also indicated flavonoids’ hepatoprotective characteristics, including better liver histology and reduced liver damage (Wu et al., 2007). Furthermore, the data from these studies reveals a probable link between flavonoid consumption and a decreased risk of HBV infection and its associated repercussions. However, translating promising findings of *in vitro* and *in vivo* studies into clinical practice remains a significant gap.

### Synergistic effects and combination therapies

Synergies between flavonoids and existing antiviral therapies, such as nucleos(t)ide analogs, have been explored. The combination of flavonoids with standard drugs may enhance antiviral efficacy, reduce drug resistance, and offer potential for more effective HBV management.

EGCG has demonstrated promising results in the combination therapy. EGCG inhibits HBV entry into hepatocytes and lowers cccDNA levels, which are required for the virus’s persistence. By limiting the establishment of cccDNA, EGCG may complement traditional anti-HBV drugs that primarily target viral replication (He et al., 2011). This synergy tackles the key difficulty in HBV treatment, the removal of viral cccDNA within the hepatocyte repositories. PAC is another promising compound, which exhibits potential for combination therapy with tenofovir, a widely used antiretroviral drug for HBV treatment (Tsukuda et al., 2017). Combining PAC with tenofovir could potentially enhance the inhibition of viral entry, making it a valuable addition to current treatment strategies.

3,5,6,7,3’,4’-Hexamethoxyflavone (Hex) is another flavonoid demonstrating potential for combination therapy. Hex reduced HBsAg levels and intracellular HBV RNA and DNA, targeting the critical steps in viral lifecycle, including antigen expression and replication (Tan et al., 2021). Therefore, combining Hex with current anti-HBV drugs may offer a comprehensive approach to reduce viral load and enhance therapeutic efficacy. Baicalin, found in Flavocoxid, presents a multifaceted approach to combat HBV infection. It inhibits HBV viral RNA, modulates NF-κB, induces proinflammatory responses to HBV, and down-regulate HNF1α and HNF4α (Chirumbolo, 2018). Flavocoxid, a baicalin-containing formula, demonstrated antiviral activity against HBV (Pollicino et al., 2018). However, further research is needed to ascertain the full potential of baicalin and flavocoxid in combination therapies.

In addition, baicalin in lipid-based nanoemulsions and hyperoside nanocrystals improve AUC and Cmax values. Higher Cmax in lymph nodes has been found to be a viable drug delivery method for CHB therapy (Shen et al., 2016; Xu et al., 2019). While this points to potential synergy with other anti-HBV drugs, especially those targeting lymphatic absorption, further research is needed to delineate the specific combinations and their extent of synergistic effects. These compounds, along with their respective biological activities, indicate their potential as valuable candidates for combination therapy in the context of HBV treatment. Such combinations could help enhance the overall effectiveness of anti-HBV drugs and address various stages of the HBV life cycle. However, it’s important to further investigate the specific drug combinations, dosage, and treatment regimens to optimize their antiviral effects. The synergy of compounds with other anti-HBV drugs offers a promising approach to enhance the effectiveness of CHB treatment, and present a fertile ground for further research and clinical trials.

### Challenges and future perspectives

**Figure 3:**
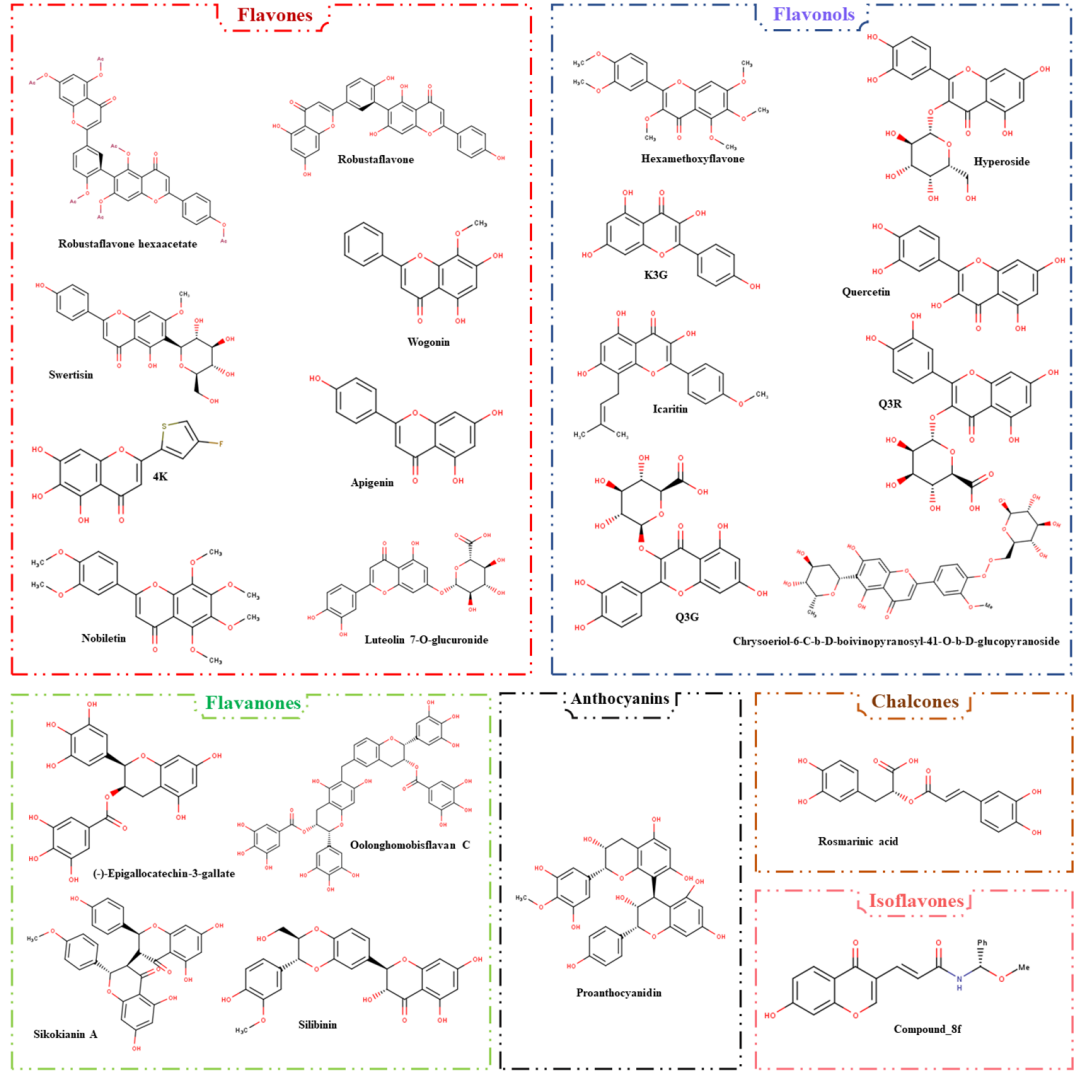
The classification of flavonoids with anti-HBV activities. The compounds are designated into six flavonoid subclasses, including flavones, flavonols, flavanones, anthocyanins, chalcones, and isoflavones.

While flavonoids have promise anti-HBV properties, different challenges must be addressed. Flavonoid molecules may have low absorption, limiting their therapeutic use. Strategies for improving their absorption and delivery are critical. Furthermore, converting *in vitro* and animal study results to human trials is a difficult undertaking that necessitates extensive clinical research. Flavonoids exert their effects through a variety of methods, making it critical to understand their specific targets and interactions with viral components. Future research is needed on well-designed clinical studies to determine the effectiveness of flavonoids as supplementary therapy for persistent HBV infections. Additionally, combination therapies with flavonoid compounds could hold the key to more effective HBV treatment strategies. Moreover, the comprehension of the structure and biological activities of anti-HBV flavonoid compounds (depicted in **Figure 3**) enables the construction of models with significant precision using computational tools and artificial intelligence, facilitating drug development research.

## Conclusion

Flavonoids, a diverse group of polyphenolic compounds found in various plant-based foods, exhibit great potential in the context of antiviral activities and particularly against HBV infection. This group of natural compounds includes different subclasses, each with unique variations and potential health benefits. Understanding the diverse classes of flavonoids and their dietary sources is crucial for harnessing their potential health benefits. In the pursuit of effective antiviral therapies against HBV, flavonoids have emerged as promising candidates. Different studies revealed the potential of flavonoids to interfere with multiple stages of the HBV life cycle, opening up avenues for novel antiviral agents. Key plant families, including *Theaceae*, *Asteraceae*, *Lamiaceae*, and *Gentianaceae*, are of particular interest, as they house plants rich in flavonoids with anti-HBV activities. The scientific exploration of these plant families involves various methods, including phytochemical analysis, biological assays, computational approaches, and clinical validation. This multidisciplinary approach is striving to advance antiviral research and provide hope for those affected by HBV infections.

Flavonoids have demonstrated diverse mechanisms of action, making them promising for HBV therapy. These mechanisms include inhibiting viral entry, disrupting viral replication, modulating transcription factors, enhancing the immune response, and inducing autophagy. Important flavonoids such as EGCG, PAC, hexamethoxyflavone, wogonin, and baicalin exhibit significant anti-HBV activity, highlighting their potential as therapeutic agents. However, it is essential to conduct further research to optimize their clinical application. Additionally, Flavonoids’ antioxidant properties play a vital role in mitigating the oxidative stress associated with HBV infection, reducing reactive ROS, and inhibiting lipid peroxidation. These effects help protect hepatocytes and liver tissues from oxidative damage, contributing to the overall reduction in liver inflammation and damage.

Experimental evidence, both *in vitro* and *in vivo*, underscores the anti-HBV potential of flavonoids. These compounds have been shown to inhibit viral replication, reduce viral DNA and antigen levels, and exhibit hepatoprotective effects in animal models. Translating these promising results into clinical practice remains a significant challenge. Also, synergistic effects between flavonoids and existing antiviral therapies have been explored, offering a potential avenue to enhance antiviral efficacy and reduce drug resistance. Flavonoids like EGCG, PAC, hexamethoxyflavone, and baicalin have shown promise in combination therapies with standard HBV drugs. These combinations could provide a more comprehensive approach to reducing viral load and enhancing therapeutic efficacy.

Above all, challenges remain, including the limited bioavailability of flavonoid and almost all other plant-derived compounds, which hinders their clinical application. Additionally, the bioactive dosage of these compounds is not adequate to exerts their anti-viral activities when consumed from the plants or fruits. Improving strategies for absorption, delivery, and dosage are essential. Additionally, translating *in vitro* and animal study results to human trials is complex and requires rigorous clinical research. Understanding the precise targets and interactions of flavonoids with viral components is crucial. Future research should focus on well-designed clinical trials, further exploration of combination therapies, and the development of flavonoid derivatives with enhanced bioavailability to optimize their effectiveness in managing chronic HBV infections. Flavonoids offer a promising avenue for the development of novel antiviral therapies against HBV, underscoring their significance in the scientific quest for effective HBV treatment. Also, integration of artificial intelligence and computer-aided drug discovery approaches for establishing reliable models based-on active flavonoids against HBV will be promising in the future studies.

## Author contributions

MN: Data curation, Formal analysis, Investigation, Writing – original draft. ZS: Visualization, Writing – original draft. UG: Data curation, Writing – review & editing. AM: Conceptualization, Project administration, Supervision, Validation, Visualization, Writing – review & editing.
